# Estimates and trends of zero vegetable or fruit consumption among children aged 6–23 months in 64 countries

**DOI:** 10.1371/journal.pgph.0001662

**Published:** 2023-06-27

**Authors:** Courtney K. Allen, Shireen Assaf, Sorrel Namaste, Rukundo K. Benedict

**Affiliations:** 1 Sociology Department, University of Washington, Seattle, Washington, United States of America; 2 The Demographic and Health Surveys Program, ICF, Rockville, MD, United States of America; African Population and Health Research Center, KENYA

## Abstract

Children require a diverse diet, that includes vegetables and fruits, to support growth and development and prevent non-communicable diseases. The WHO-UNICEF established a new infant and young child feeding (IYCF) indicator: zero vegetable or fruit (ZVF) consumption among children aged 6–23 months. We estimated the prevalence, trends, and factors associated with ZVF consumption using nationally representative, cross-sectional data on child health and nutrition in low-and-middle-income countries. We examined 125 Demographic and Health Surveys in 64 countries conducted between 2006–2020 with data on whether a child ate vegetables or fruits the previous day. Prevalence of ZVF consumption was calculated by country, region, and globally. Country trends were estimated and tested for statistical significance (p<0.05). Logistic regression analysis was used to examine the relationship between ZVF and child, mother, household, and survey cluster characteristics by world region and globally. Using a pooled estimate of the most recent survey available in each country, we estimate the global prevalence of ZVF consumption as 45.7%, with the highest prevalence in West and Central Africa (56.1%) and the lowest in Latin America and the Caribbean (34.5%). Recent trends in ZVF consumption varied by country (16 decreasing, eight increasing, 14 no change). Country trends in ZVF consumption represented diverse patterns of food consumption over time and may be affected by the timing of surveys. Children from wealthier households and children of mothers who are employed, more educated, and have access to media were less likely to consume ZVF. We find the prevalence of children aged 6–23 months who do not consume any vegetables or fruits is high and is associated with wealth and characteristics of the mother. Areas for future research include generating evidence from low-and-middle-income countries on effective interventions and translating strategies from other contexts to improve vegetable and fruit consumption among young children.

## Introduction

Vegetable and fruit consumption is an important part of a healthy diet. From age 6 months, children require more than breastmilk alone to meet their nutritional needs as they experience rapid growth and development in the first two years of life [1, 2]. Young children are at risk of malnutrition as they transition from only breastmilk to consuming family foods in addition to breastmilk. It is vital to introduce appropriate complementary feeding practices with diverse, nutrient-rich foods that include vegetables and fruits [1]. The intake of vegetables and fruits by infants and young children protects against growth faltering, influences eating behaviors later in life, and has long-term protective effects against non-communicable diseases [3–6].

The consumption of vegetables and fruits is a component of minimum dietary diversity (MDD) and a recent study of 80 low-and-middle-income countries (LMIC) found that MDD among children aged 6–23 months is low (globally, 27.1%) and improves with income [7]. Other studies examining factors associated with MDD have identified child age, maternal age, employment, education, access to media, and household wealth as important factors [8–11]. Data on vegetable and fruit consumption specifically, have predominately been published in high-and-middle-income countries [12–15]. In the United States, the 2016 Feeding Infants and Toddlers Study reported that based on a 24-hour recall, nearly three-quarters of children aged 6–23 months consumed vegetables and fruits on the day of interview [12]. Studies that used 24-hour recall data from the 2012 Mexican National Health and Nutrition Survey report that 60–87% of children aged 6–23 months consumed vegetables and fruits, but consumption of vegetables and fruits were not among the top contributors for micronutrient and fiber intake in young children’s diets [14, 15]. In China, authors reported suboptimal vegetable and fruit consumption among children aged 6–23 months in the Maternal Infant Nutrition Growth Study [13]. Data from low-income settings have traditionally been scarce. Two studies from the Philippines reported that in a 24-hour recall, few children aged 6–23 months consumed vegetables (11% to 30%) and fruits (5% to 14%), and consumption varied by income [16, 17]. More recently a multi-country study on vegetable and fruit consumption among children 6–23 months reported an overall prevalence of zero fruit or vegetable consumption in low-income and lower-middle-income countries of 45%. The study found that vegetable and fruit consumption was higher in wealthier countries and that overall poorer and rural children consumed less fruits and vegetables than their counterparts [18].

Infant and young child feeding (IYCF) indicators capture optimal feeding practices that target children during their vulnerable growth periods. The World Health Organization (WHO) and United Nations Children’s Fund (UNICEF) recently updated the IYCF indicators and a new indicator was established: zero vegetable or fruit (ZVF) consumption among children aged 6–23 months [19]. This indicator measures no vegetable or fruit consumption during the previous day or night. This information can be used to monitor and track country and global progress and inform policy and programs to promote healthy eating practices among young children.

This paper describes prevalence estimates and trends of ZVF consumption among children aged 6–23 months in low-and-middle-income countries. Using available Demographic and Health Survey (DHS) data we present prevalence estimates of ZVF consumption at the country, world region, and global levels and show the trends in ZVF consumption by country. We add to previous knowledge by investigating the relationship between ZVF consumption and characteristics relating to the mother, child, household, and cluster grouped by world regions. To our knowledge, this is the first study to present ZVF consumption trends over time.

## Methods

### Data

We use nationally representative, cross-sectional data on vegetable and fruit consumption from 125 Demographic and Health Surveys in 64 countries conducted between 2006 and 2020 (see Tables A-E in S1 Appendix). These surveys utilize a two-stage sampling approach that is designed to obtain data that are representative at the national, regional, and residence (urban-rural) level within each country. DHS surveys use a list-based recall approach to collect information from caregivers about foods fed to young children. An interviewer reads out a list of foods to the respondent, usually the parent or caregiver of the child in question, who confirms whether the child did or did not eat the food during the previous day or night before the interview. These questions typically include five questions listing different types of vitamin A-rich fruits and vegetables and any other fruits and vegetables consumed–the food items listed vary depending on local dietary patterns. Children who only consumed other plant-based foods, roots, grains, or cereals are not captured by this indicator. The consumption of ZVF is defined as the percentage of children 6–23 months of age who did not consume any vegetables or fruits during the previous day or night before the interview [19]. Cases with missing data for fruit and vegetable consumption were few (see Tables A-E in S1 Appendix) and were included in the denominator of this indicator only.

The DHS datasets also provide child, mother, and household or cluster-related characteristics that we include in our analysis based on previous studies [8–11, 20]. These are child’s age in months, sex, birth order (grouped as first born, second to fourth born, or higher order birth), and breastfeeding status (currently breastfeeding versus not); mother’s current age (ages grouped as less than 24, 25–34, 35 and older), education (none or primary versus secondary or higher), employment status (employed or not employed), media exposure in the last week to radio, television, or newspaper (any exposure versus no exposure); and place of residence (urban or rural) and household wealth quintile (lowest to highest). The household wealth quintile is a measure of economic status by accounting for household characteristics and assets and access to infrastructure and services in rural and urban areas separately. These assets are analyzed through a principal component analysis and the results are analytically combined to represent a nationally applicable wealth score ranking each household. Each household member in the surveys is assigned their household wealth score and divided into quintiles from the lowest (i.e. poorest) quintile to the highest (i.e. wealthiest) quintile and made available in all DHS datasets [21, 22]. DHS datasets are available for public use. All interviews are conducted with the verbal consent of all participants and DHS datasets are anonymized to protect the privacy of participants. The Institutional Review Board of ICF, along with relevant in-country ethical boards, reviewed and approved the survey protocols and questionnaires used during the collection of DHS Program data. The Institutional Review Board of ICF complied with the United States Department of Health and Human Services regulations for the protection of human research subjects (45 CFR 46).

To capture the time of year that countries may experience lean seasons of food availability which typically coincides with the rainy season, a binary variable was constructed to reflect whether the interview took place during the rainy season. Using a similar approach as Thorne-Lyman and colleagues [20], we use monthly estimates of precipitation provided by the Global Precipitation Climatology Project at the University of Maryland [23] to construct rainfall maps for the 12-month period preceding the last month of interview of the most recent survey. Interview months were coded as either part of the rainy season or not.

Additionally, mean temperature, enhanced vegetation index (EVI), and elevation of the survey clusters, were included. The EVI measures the amount of vegetation in an area and was standardized due to its large range of values. Mean temperature and EVI are collected every five years and were matched to the closest year of data available. Temperature, enhanced vegetation index, and elevation have been linked to agriculture and proximity to agricultural centers which may affect food availability [24–27]. These data use external geospatial data sources that are linked to the DHS survey cluster, or primary sampling unit using GPS coordinates [28].

### Data analysis

For the analyses, countries were categorized into major regions similar to those of the World Health Organization. For sub-Saharan Africa, there were many surveys available, and were grouped into two subregions. The regions are West and Central Africa (WCAFR), South and East Africa (SEAFR), Eastern Mediterranean and Europe (EMD-EUR), South-East Asia and Western Pacific (SEA-WP), and Latin America and the Caribbean (LAC).

We estimated the prevalence of ZVF consumption among children aged 6–23 months by country, world region, and globally. These analyses used the most recent survey available in each country (Table 1). Trend analyses included countries with more than one survey from 2006 to 2020 in order to examine recent trends. Additionally, most surveys since 2006 collected nutrition information per the IYCF indicator guidelines. For countries with continuous DHS surveys every year in this period, non-consecutive surveys were selected for the analysis. The difference in ZVF consumption between consecutive surveys and between the first and last survey was calculated using t-tests. Trends were considered significant with a p-value less than p<0.05. A reliable global or regional trend could not be produced due to variation in number of surveys and time between surveys in each country.

**Table 1:** Description of data included in each analysis by world region.

	Prevalence Estimates^1,2^			Trends analysis		Multivariate analysis		
Region	Survey year range	Total countries	Weighted N^3^	Survey year range	Total countries	Survey year range	Total countries	Weighted N^3^
West and Central Africa	2010–2019	19	60,317	2006–2019	10	2010–2020	17	54,414
South and East Africa	2011–2019	14	34,571	2006–2019	11	2011–2019	14	35,985
Eastern Mediterranean and Europe	2012–2018	8	25,154	2007–2018	5	2012–2018	6	12,338
South-East Asia and Western Pacific	2014–2018	10	94,842	2005–2018	8	2014–2018	7	86,190
Latin America and the Caribbean	2009–2017	7	18,124	2005–2017	4	2009–2017	6	15,360
**Global**	**2009–2020**	**58**	**235,091**	**NA**	**NA**	**2009–2020**	**50**	**204,477**

Note: NA-Not Applicable

1 While the prevalence of ZVF consumption for the most recent survey in each of the 64 study countries is shown in Tables F-J in S1 Appendix, only the 58 recent surveys since 2009 were included in each regional group prevalence.

2 Survey years included in this table are the year the survey was completed, though some surveys span two calendar years (e.g., 2005–2006).

3 Weighted N uses pooled and denormalized weights, so each country contributes equal weighting to grouped analysis

Finally, we used bivariate and multivariate logistic regression to examine factors associated with ZVF consumption in each region and globally. The analysis pooled the most recent survey in each country from 2009 that collected geospatial coordinates and had geospatial covariate datasets available (Table 1). Covariates were tested for correlation and none were correlated. Variable selection for the models was theoretically driven and covariates were included in logistic regression models based on conceptual relevance regardless of the p-value. The models controlled for child, mother, and household characteristics. Additionally, we include cluster fixed effects for the rainy season, month of interview, and country. An interaction term between the child’s age in months and breastfeeding status was also included.

All analyses considered the sampling procedures used in the surveys by accounting for the primary sampling unit, stratification, and sampling weights and these elements were incorporated using the *svy* command in Stata. For each survey, we applied survey weights to account for the 2-stage cluster sampling design of the surveys, and for the regional and global analyses, data were pooled and survey sampling weights from country surveys were denormalized, so each country contributed equal weighting to group analysis. Cases with missing data for any variables included in the model were excluded using listwise deletion. Stata version 17 was used for data manipulation and analysis and R version 4.1.1 was used to produce figures.

## Results

### Zero vegetable or fruit consumption prevalence

**Fig 1:**
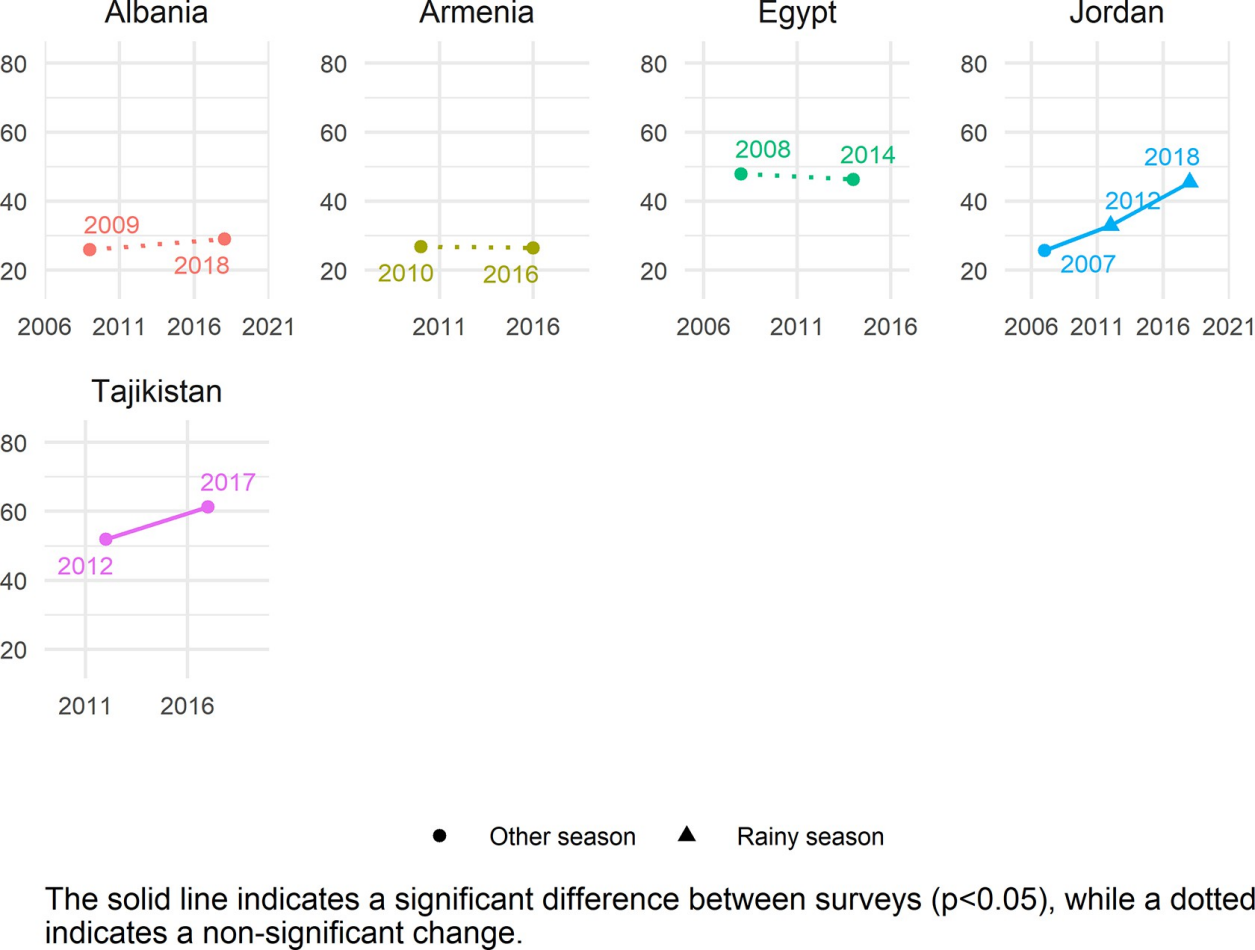
Trends in zero vegetable or fruit consumption among children aged 6–23 months for countries in EMD-EUR.

**Fig 2:**
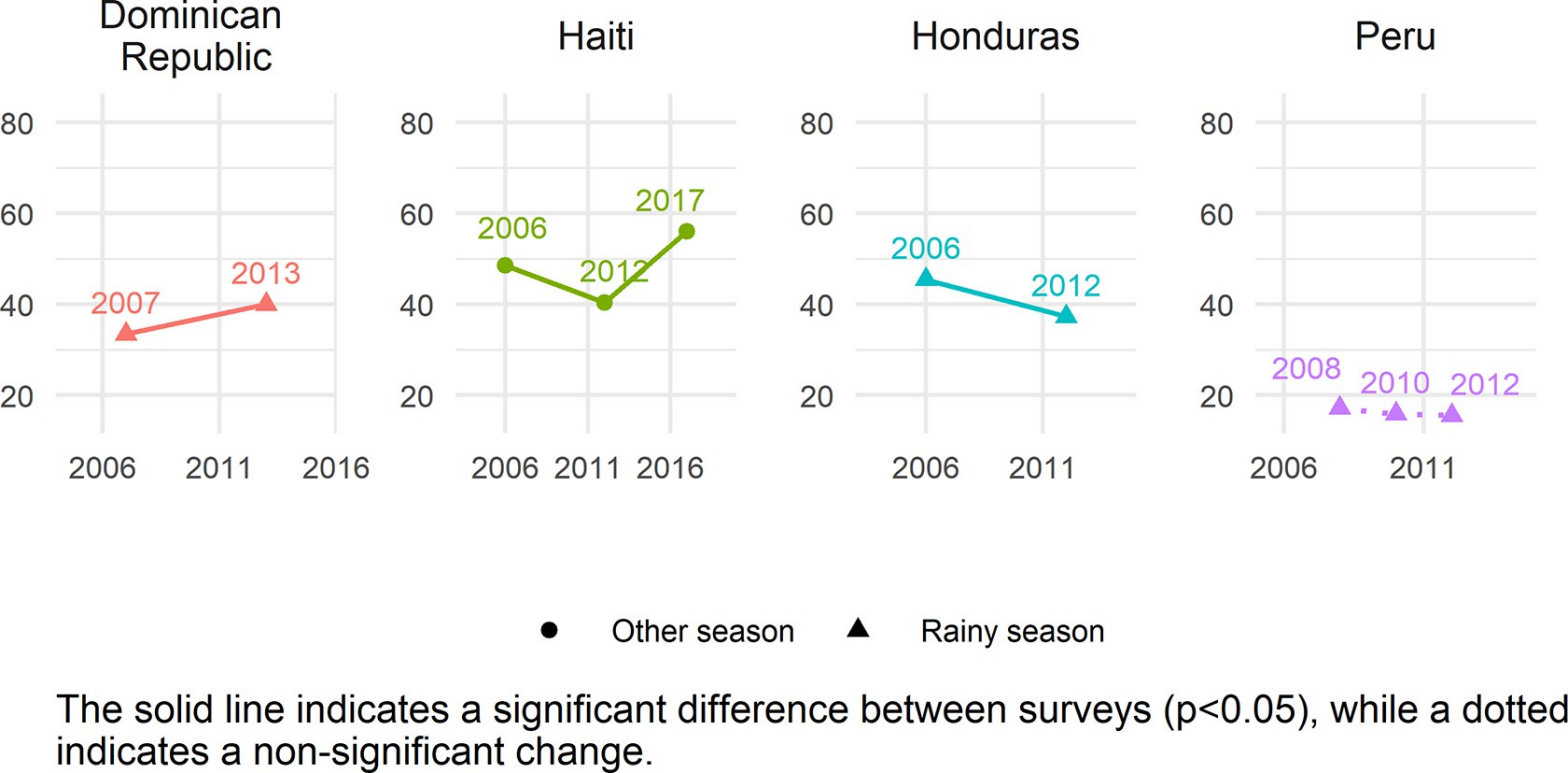
Trends in zero vegetable or fruit consumption among children aged 6–23 months for countries in LAC.

**Fig 3:**
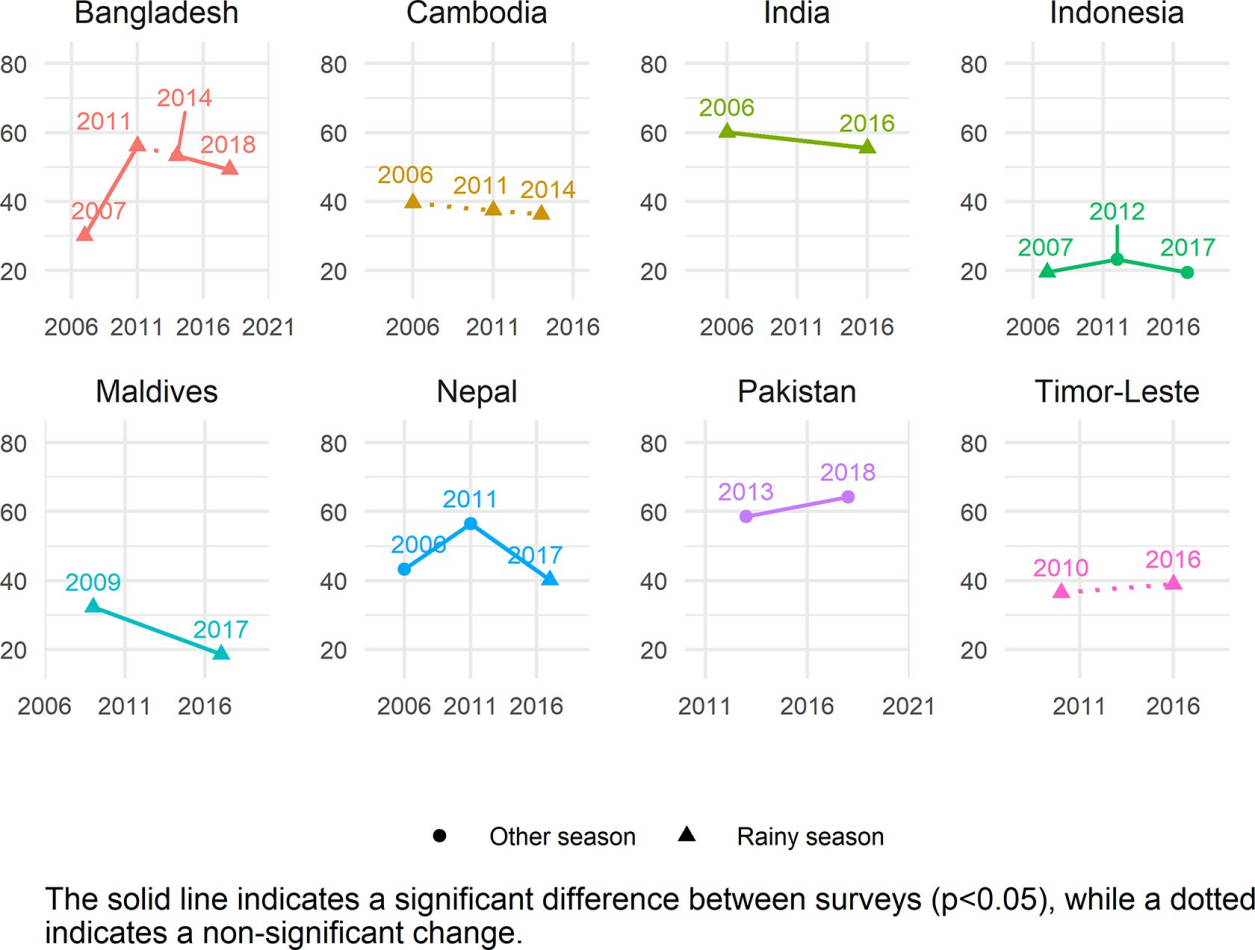
Trends in zero vegetable or fruit consumption among children aged 6–23 months for countries in SEA-WP.

**Fig 4:**
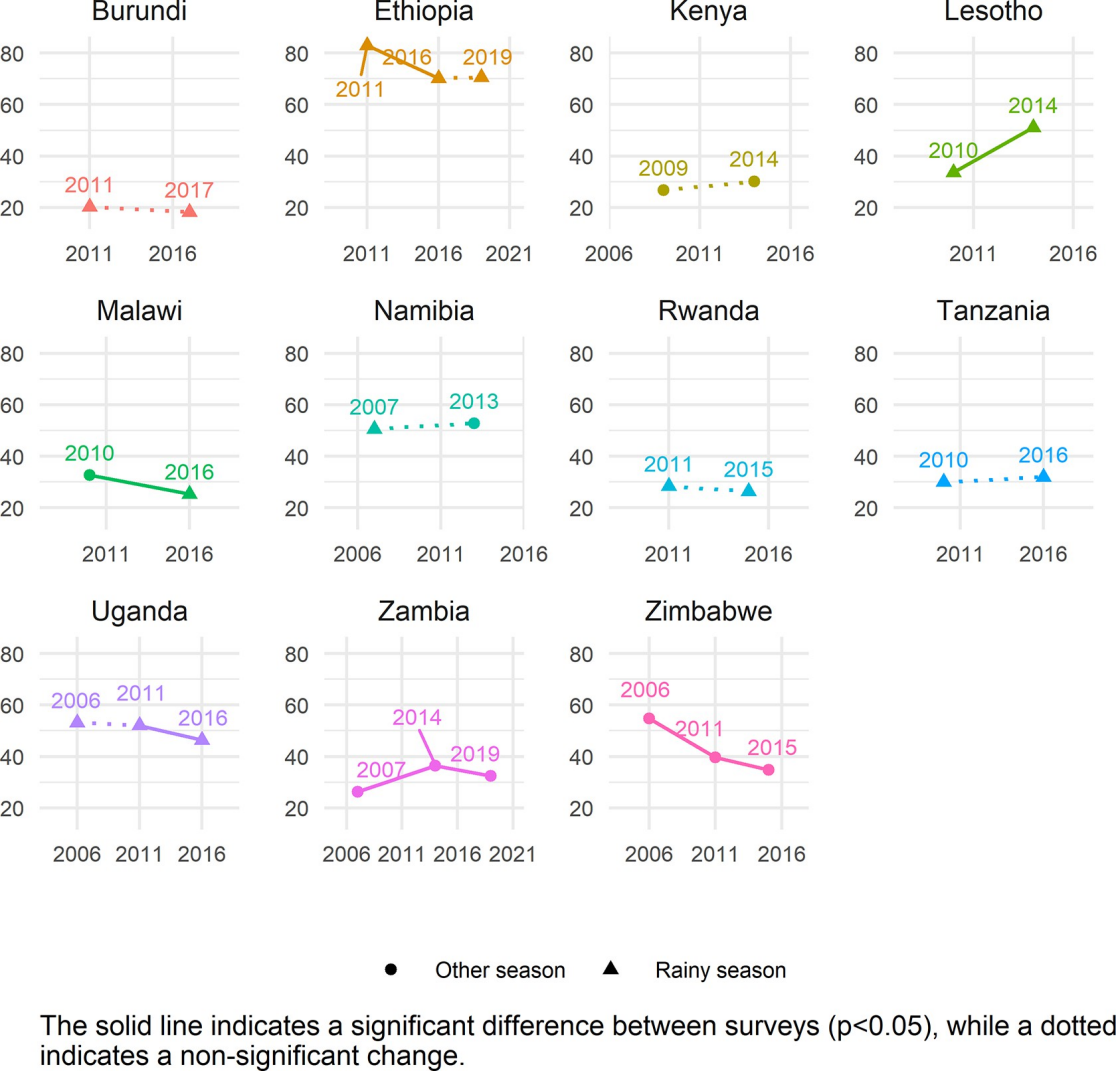
Trends in zero vegetable or fruit consumption among children aged 6–23 months for countries in SEAFR.

**Fig 5:**
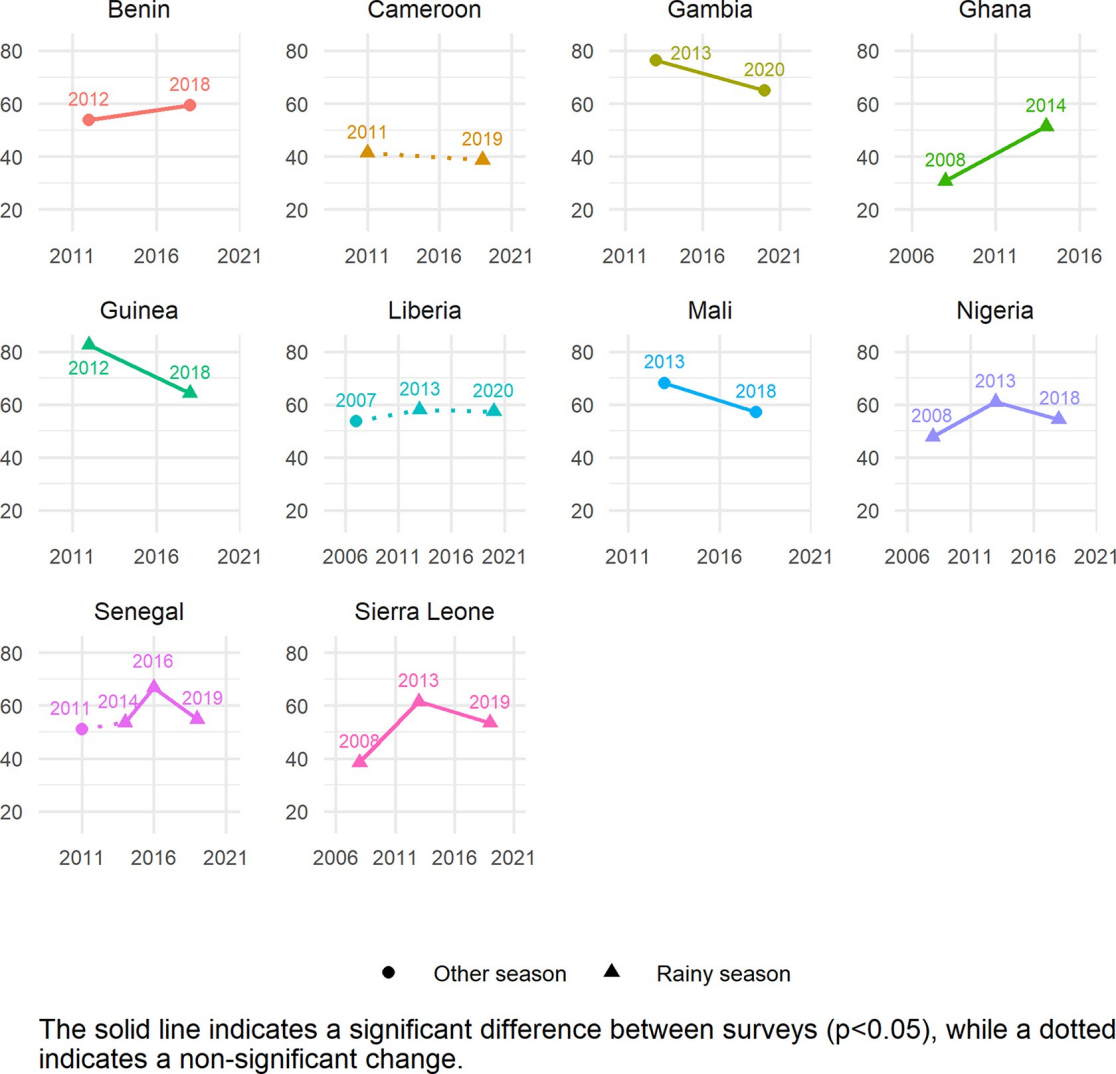
Trends in zero vegetable or fruit consumption among children aged 6–23 months for countries in WCAFR.

The global estimate of ZVF consumption was 45.7% (95% CI 45.2–46.2). Between 34.5–56.1% of children aged 6–23 months from all world regions consumed ZVF in the previous day (Table 2). The two regions with the highest ZVF consumption were WCAFR (56.1% [95% CI 55.4–56.9]) and EMD-EUR (48.3% [95% CI 46.9–49.7]). The lowest prevalence of children with ZVF consumption was in LAC (34.5% [95% CI 33.3–35.7]). Season of data collection only varies in some countries (season shown in Figs 1–5), and there is no clear relationship between season of data collection and ZVF consumption.

**Table 2:** Percentage of children aged 6–23 months with zero vegetable or fruit consumption, by world region.

Region	Year Range	Prevalence (%)	95% CI	Weighted N
West and Central Africa	2010–2020	56.1	[55.4–56.9]	60,317
South and East Africa	2011–2019	39.5	[38.4–40.5]	34,571
Eastern Mediterranean and Europe	2012–2018	48.3	[46.9–49.7]	25,154
South-East Asia and Western Pacific	2014–2018	39.7	[38.6–40.7]	94,842
Latin America and the Caribbean	2009–2017	34.5	[33.3–35.7]	18,124
**Global**	**2009–2020**	**45.7**	**[45.2–46.2]**	**235,091**

Note: CI-Confidence Interval

By country, there was high variability in the percentage of ZVF consumption (see Tables F-J in S1 Appendix). Cote d’Ivoire (76.2% [95% CI 73.6–78.6]) had the highest percentage of ZVF consumption followed by Burkina Faso (75.0% [95% CI 73.1–76.7]). There was low prevalence in Peru (15.6% [95% CI 13.9–17.4]), Papua New Guinea (17.7% [95% CI 15.2–20.2]), Burundi (18.2% [95% CI 16.8–19.8]), Maldives (18.7% [95% CI 14.8–23.2]), and Indonesia (19.5% [95% CI 18.1–20.8]). There was also variability in ZVF consumption within world regions, with the greatest range in SEAFR (ranged from 18.2% in Burundi (95% CI 16.8–19.8) to 70.5% in Ethiopia (95% CI 65.8–74.9)).

### Country trends

Figs 1–5 show the trends in ZVF consumption between the surveys for each country by region (see also Table K in S1 Appendix) and timing of the survey i.e., during the rainy season or other season. A decrease in ZVF consumption indicates an improvement, i.e., fewer children over time have not consumed any vegetables or fruits the day before the survey. Overall, among the 38 countries included in the trends analysis, 16 had significant decreases in ZVF consumption between the last two surveys, eight had significant increases, and 14 showed no significant change. Across countries, there were no clear patterns observed between ZVF consumption and timing of the survey.

Among the five countries in EMD-EUR, we see no significant change in ZVF consumption in Armenia, Albania, and Egypt (Fig 1) and all surveys were conducted during a non-rainy season. There was a continued increase in ZVF consumption in Jordan during the two most recent surveys which were conducted in the rainy season, reaching nearly half of children (45%). There was an increase in Tajikistan from 52% to 61%, the highest prevalence in this region and both surveys were conducted during a non-rainy season.

In LAC, Peru was the only country with no significant change between the two most recent surveys, and all surveys were conducted in a rainy season. Peru is the country with the lowest percentage of ZVF consumption (< 20% in both surveys, see Fig 2). The percentage of children with ZVF consumption decreased in Honduras (both surveys conducted in the rainy season) but increased in the Dominican Republic (both surveys conducted in the rainy season) and Haiti (all surveys conducted in a non-rainy season). Haiti increased from 40% to 56% between the two most recent surveys.

In SEA-WP, Bangladesh, Nepal, and Indonesia had significant increases in ZVF consumption followed by a significant decrease between the two most recent surveys (Fig 3). Nepal’s most recent survey was the only survey conducted during the rainy season and only Indonesia’s last two surveys were conducted during the non-rainy season. All other countries in the region conducted their surveys during the rainy season, with the exception of Pakistan’s survey being conducted in the non-rainy season. Significant decreases overall occurred in the percentage of children who consumed ZVF in India and the Maldives. The most recent surveys in Indonesia and the Maldives had the lowest percentage of ZVF consumption in this region (20% and 19% respectively). While there was a decrease in ZVF consumption in India, it remains one of the highest in the region with over half of children (56%) consuming ZVF in the most recent survey.

In SEAFR, there was a large increase in ZVF consumption among children Lesotho (18 percentage point increase) (Fig 4). The percentage of ZVF consumption between the two most recent surveys decreased in Uganda, Zambia, and Zimbabwe. In Ethiopia there was a decrease in ZVF consumption between earlier surveys and then no significant change between the two most recent surveys; Ethiopia remains the country with the highest percentage of ZVF consumption in this region (71%). Surveys were conducted in the same season within countries, with the exception of Malawi and Namibia. Malawi saw significant decrease in ZVF, and the latter survey was the only survey conducted in the rainy season. Namibia’s second survey was the only survey within the country to be conducted in the non-rainy season, and there was no significant change detected in ZVF consumption between surveys.

In WCAFR, seven countries showed significant decreases in ZVF consumption (Gambia, Guinea, Mali, Nigeria, Senegal, Sierra Leone) (Fig 5). Ghana and Benin had a significant increase, and there were no significant differences in Cameroon and Liberia between the two recent surveys. For Nigeria, Senegal, and Sierra Leone, there was a significant increase in ZVF consumption between earlier surveys, followed by a significant decrease between the two recent surveys. Guinea had the greatest improvement in ZVF consumption decreasing from 83% to 64%. Changes in season of data collection had mixed results in direction of ZVF consumption.

### Regional and global multivariate analysis

Table 3 summarizes the regression results for the global model and each world region.

**Table 3:** Multivariate regression results with adjusted odds ratios and 95% confidence intervals for the global and regional models.

	Global		West & Central Africa		South & East Africa		Eastern Mediterranean & Europe		South-East Asia & Western Pacific		Latin America & the Caribbean	
	AOR	95% CI	AOR	95% CI	AOR	95% CI	AOR	95% CI	AOR	95% CI	AOR	95% CI
**Child’s characteristics**												
Birth order												
First born (ref)	..	..	..	..	..	..	..	..	..	..	..	..
Second—fourth	0.98	0.93–1.02	0.99	0.91–1.07	1.03	0.94–1.14	0.82**	0.71–0.95	0.96	0.87–1.05	1.03	0.89–1.18
Fifth +	1.00	0.94–1.07	0.99	0.89–1.09	1.00	0.88–1.13	0.87	0.67–1.12	1.05	0.89–1.24	1.24	1.00–1.55
Sex of child												
Male (ref)	..	..	..	..	..	..	..	..	..	..	..	..
Female	0.98	0.95–1.02	1.01	0.96–1.07	0.93*	0.86–0.99	0.95	0.84–1.08	0.98	0.90–1.08	1.03	0.92–1.15
Age	0.95***	0.94–0.96	0.93***	0.92–0.95	0.96***	0.95–0.98	0.95***	0.93–0.97	0.93***	0.91–0.94	0.96***	0.94–0.98
Breastfeeding												
Not currently (ref)	..	..	..	..	..	..	..	..	..	..	..	..
Currently	2.50***	2.13–2.93	2.12***	1.64–2.75	2.88***	2.04–4.05	2.91***	1.81–4.66	2.19***	1.54–3.12	1.79**	1.22–2.62
Breastfeeding x Age Interaction	0.94***	0.93–0.95	0.95***	0.94–0.96	0.93***	0.91–0.95	0.93***	0.90–0.96	0.95***	0.93–0.97	0.95***	0.93–0.97
**Mother’s characteristics**												
Education												
None, primary (ref)	..	..	..	..	..	..	..	..	..	..	..	..
Secondary, higher	0.82***	0.78–0.86	0.87***	0.80–0.93	0.87**	0.80–0.96	0.70***	0.57–0.86	0.74***	0.67–0.82	0.83**	0.72–0.95
Employment												
Not working (ref)	..	..	..	..	..	..	..	..	..	..	..	..
Working	0.79***	0.76–0.83	0.83***	0.78–0.88	0.75***	0.69–0.81	0.85	0.71–1.03	0.85**	0.76–0.95	0.80***	0.71–0.90
Media exposure												
None (ref)	..	..	..	..	..	..	..	..	..	..	..	..
Some	0.88***	0.84–0.92	0.93*	0.87–1.00	0.89**	0.83–0.96	0.71**	0.56–0.90	0.82***	0.74–0.92	0.75***	0.63–0.89
Mother’s age												
<24 (ref)	..	..	..	..	..	..	..	..	..	..	..	..
25–24	0.98	0.92–1.05	0.97	0.88–1.08	1.01	0.88–1.15	1.11	0.80–1.53	0.95	0.80–1.13	0.89	0.72–1.08
35+	0.92	0.85–1.01	0.91	0.80–1.03	0.93	0.79–1.10	1.10	0.77–1.59	0.93	0.74–1.16	0.82	0.62–1.09
**Household & cluster characteristics**												
Wealth												
Lowest (ref)	..	..	..	..	..	..	..	..	..	..	..	..
Second	0.93**	0.89–0.98	1.00	0.93–1.08	0.93	0.84–1.02	0.92	0.78–1.08	1.04	0.92–1.18	0.84*	0.72–0.98
Middle	0.87***	0.82–0.92	0.93	0.85–1.01	0.86**	0.77–0.96	0.82*	0.69–0.98	1.00	0.87–1.15	0.77**	0.64–0.92
Fourth	0.82***	0.77–0.87	0.94	0.85–1.04	0.81***	0.72–0.91	0.75**	0.61–0.91	0.95	0.82–1.10	0.59***	0.47–0.73
Highest	0.70***	0.65–0.76	0.81**	0.71–0.93	0.74***	0.63–0.87	0.60***	0.46–0.78	0.82*	0.69–0.98	0.42***	0.33–0.54
Residence												
Urban (ref)	..	..	..	..	..	..	..	..	..	..	..	..
Rural	1.03	0.98–1.09	1.05	0.96–1.15	1.18**	1.05–1.33	0.89	0.74–1.07	1.06	0.94–1.19	0.93	0.81–1.08
Rainy season												
No (ref)	..	..	..	..	..	..	..	..	..	..	..	..
Yes	1.05	0.99–1.12	0.95	0.82–1.10	1.11	0.98–1.26	0.86	0.60–1.23	1.46**	1.17–1.83	0.81	0.64–1.03
Mean temperature	1.00	1.00–1.00	1.00	1.00–1.00	1.00**	1.00–1.00	1.00**	1.00–1.00	1.00	1.00–1.00	1.00	1.00–1.00
Vegetation index ^1^	0.97**	0.95–0.99	0.97	0.93–1.01	0.94**	0.90–0.98	1.01	0.94–1.09	0.95	0.90–1.00	0.98	0.92–1.04
Altitude ^2^	1.00*	1.00–1.00	1.00	1.00–1.00	1.00***	1.00–1.00	1.00**	1.00–1.00	1.00	1.00–1.00	1.00	1.00–1.00

Note: Month and country was also included as a fixed effect. Asterisks indicate the p-value *p<0.05, **p<0.01, ***p<0.001. AOR—Adjusted Odds Ratio. CI—Confidence Interval. ‘Ref’–reference category. 1 Unitless index. Higher scores indicate higher vegetation vigor/photosynthetic activity, 2 Degrees of elevation

### Children’s characteristics

Among the child’s characteristics, the interaction term between breastfeeding and age was significant in all regions with minimal differences between the regions and a global AOR of 0.94 (95% CI 0.93–0.95). This indicates that as age increases, ZVF consumption decreases among breastfed children compared to non-breastfed children. The birth order of the child was only significantly associated with ZVF consumption in EMD-EUR (second to fourth born compared to first born AOR 0.82 [95% CI 0.71–0.95]) and child’s sex was only significantly associated with ZVF consumption in SEAFR (female compared to male AOR 0.93 [95% CI 0.86–0.99]).

### Mother’s characteristics

Among mothers’ characteristics, children with mothers that have secondary or more education level had significantly lower odds of ZVF consumption compared to mothers that have below secondary education (global AOR 0.82 [95% CI 0.78–0.86]). This was also significant across all regions with the lowest odds ratio in EMD-EUR, where the odds of ZVF consumption for children with mothers that have secondary or more education were 30% lower compared to mothers with below secondary education (AOR 0.70 [95% CI 0.57–0.86]). Children of employed mothers were significantly less likely to consume ZVF compared to unemployed mothers in the global model and all regions except EMD-EUR. The significant AORs ranged from 0.75 (95% CI 0.69–0.81) in SEAFR to 0.85 (95% CI 0.76–0.95) in SEA-WP and a global AOR of 0.79 (95% CI 0.76–0.83). The mother’s exposure to media messages also resulted in lower odds of ZVF compared to those with no media exposure and was significant in all regions. The global AOR was 0.88 (95% CI 0.84–0.92) and ranged from 0.71 (95% CI 0.56–0.90) in EMD-EUR to 0.93 (95% CI 0.87–1.00) in WCAFR.

### Household and cluster characteristics

Among household and other cluster characteristics, the relationship between ZVF consumption and wealth quintile differed by region. Globally, children in the highest wealth quintile have 30% lower odds of ZVF consumption than from the lowest wealth quintile (AOR 0.70 [95% CI 0.65–0.76]). In the global model, SEAFR, EMD-EUR, and LAC, middle and higher categories of wealth had lower odds of ZVF consumption, while in WCAFR and SEA-WP lower odds of ZVF consumption were only found in the highest quintile compared to the lowest. The disparities by wealth were the largest in LAC with children in the highest wealth quintile having 58% lower odds of ZVF consumption than children in the lowest wealth quintile (AOR 0.42 [95% CI 0.33–0.54]).

Rural dwellers were also more likely to consume ZVF than urban dwellers in SEAFR (AOR 1.18 [95% CI 1.05–1.33]), while urban or rural residence had no significant association in any other region. We also observe few significant findings among seasonality and climate-related variables. The rainy season was statistically significantly with higher odds of ZVF consumption in SEA-WP (AOR 1.46 [95% CI 1.17–1.83]). Mean temperature, vegetation index, and altitude were statistically significant in some models (global, SEAFR and EMD-EUR), but with odd ratios close to or one, indicating a very small effect to no effect and suggesting that these relationships are not meaningfully significant.

## Discussion

We examined zero vegetable or fruit consumption among children aged 6–23 months using 129 DHS surveys from 64 countries from 2006 to 2020. Globally, approximately half of children (45.7%) had not consumed any fruits or vegetables in the previous day before the survey and these estimates are in line with other research that has estimated global ZVF consumption in LMICs at 45% [18]. Regionally, LAC had the lowest prevalence of ZVF consumption (34.5%), while WCAFR had the highest (56.1%). Among the 40 countries included in the trends analysis, 17 had significant decreases in ZVF consumption between the last two surveys, 9 had significant increases, and 14 showed no significant change. In the multivariate models, wealth and mother’s characteristics had the lowest odds of ZVF consumption.

Consistent with recent research on estimates of minimum dietary diversity and minimum acceptable diet we found regional variations in the prevalence of ZVF consumption. These studies also identify the LAC and WCAFR regions as having the highest and lowest diet diversity, respectively, illustrating that our results align with those related to other infant feeding indicators [7, 29]. Though studies often examine sub-Saharan Africa as one region, we showed important sub-regional differences in ZVF consumption (WCAFR 56.1% and SEAFR 42.0%).

The trend analyses showed inconsistent patterns. In WCAFR and SEA-WP, most but not all countries exhibited significant improvements i.e., declining rates of ZVF. In SEAFR results were mixed and in EMD-EUR trends were mostly stable. The reasons for the variability of ZVF consumption patterns within countries is unknown. It could be explained by changes in food insecurity, agriculture production, or dietary patterns [27], though large fluctuations in ZVF may be unlikely and due to other sources of measurement error. DHS surveys take several months and span growing seasons, which may coincide with the seasonality of available fresh vegetables and fruits. This means that within surveys, variation in ZVF consumption may be due to seasonal food availability. To this end, surveys would need to be conducted across different seasons (rainy vs. non-rainy) within the same country and year to understand increased or diminished seasonal availability of fruits and vegetables. In general, care should be taken when interpreting trends as although standardized questionnaires are used, questions can be modified between surveys and language translations may also contribute to measurement error. While our study is the first to illustrate trends in ZVF consumption, further analyses of country trends are required to better understand these patterns and monitor progress.

Our study shows that wealthier households and children whose mothers had more education, exposure to media, or were employed had lower odds of ZVF consumption. These findings are similar to other studies that have examined MDD [7–11, 17] and fruit and vegetable consumption [18] in children. Additionally, data from high-income settings describe a range in the prevalence fruit and vegetable consumption that is similar to some of the LMICs included in our study and this may suggest similar underlying socioeconomic inequalities that contribute to ZVF consumption across contexts [12–15]. Behavioral change interventions in low-and middle-income countries have been found to improve complementary feeding practices and the quality of children’s diets, but vegetable and fruit consumption was not a specific outcome in these studies [5, 30, 31]. Ultimately, behavior change in food utilization are preceded by food availability and access. An enabling environment can be achieved through policy changes, such as taxation of less healthy foods and subsidizing healthier food or other policies to help mitigate poor households experiencing sharp increases in food costs due to economic shocks [32–34]. Conditional cash transfer and food assistance programs can also provide direct support to less wealthy families and have been shown to improve fruit and vegetable consumption among children [30, 31].

The interaction term in our analysis shows that as children age, those who breastfed have lower odds of ZVF consumption than those who are not breastfed. One explanation is that breastfeeding infants may have a delayed introduction to complementary foods and younger children are not consuming any complementary foods compared to non-breastfed children. Cereal and grain-based foods are common complementary foods and because non-breastfeeding children may have been introduced to these foods earlier, they are more likely to continue consuming these types of foods [7]. Lastly, there is evidence that breastfeeding infants and young children whose mothers consume vegetables and fruits have a higher exposure to the variety of flavors from their mother’s diet via breast milk and this may potentially may increase children’s acceptance of new foods [35].

Interestingly, the season or climate did not have a strong association with children’s vegetable and fruit consumption across world regions. The rainy season variable was only associated with higher ZVF consumption in SEA-WP, while the other climatic variables showed weak to null associations in SEAFR and EMD-EUR. Seasonality is likely to affect both the availability and price of fruits and vegetables, with those in rural and subsistence farming areas especially vulnerable. As the association between rural residence and higher ZVF consumption in SEAFR suggests. The lack of a strong association with the seasonality and climatic variables in our study may be due to several reasons. First, the classification threshold for ZVF consumption indicator is low and a child is not classified as having ZVF consumption if fed just one vegetable or fruit. It may be that season or climate has less impact on ZVF consumption, even though the amount of vegetables and fruits being consumed may vary due to food availability throughout the year [20]. This limitation means the ZVF indicator cannot detect variation in the amount of fruit and vegetable consumption. It only captures that the consumption is or is not zero. In addition, our indicators for season and climate may not be sufficiently robust. For example, the rainy season variable is a proxy for lean season [36] and countries are likely to experience more than one lean season in a year; moreover, many surveys in our analysis collected data for six months or longer, which may overlap several seasons and add to the complexity in interpreting the trend results. Within a country there may be seasonal variation and different climatic regions which affect food availability. Even though our regional and global models controlled for country, there is likely to be some variation that our models and our seasonal measure cannot capture. The importance and magnitude of the effect that climate and season play on the availability of fresh vegetables and fruits for consumption among young children warrants further study.

Our study is not without other limitations. The analyses were also limited to the global, regional, or country level for regressions and trends, respectively. However, there is large variability within countries due to environmental, social, economic, and other factors that can influence ZVF consumption. Therefore, the analyses could mask national or subnational complexities related to ZVF consumption. In addition, due to data availability, some regional categories did not include many countries. For this reason, generalization to the regional level should be done with caution, especially for the non-African regions where fewer countries were included. Another limitation is the ZVF consumption indicator, which is based on the mother’s report of the foods her child consumed in the day or night before the survey and may not reflect a child’s usual diet and is prone to both under and overreporting [37]. Overreporting of vegetables and fruits may occur if items consumed in insufficient quantities are reported, or due to social desirability bias. Conversely, underreporting may occur if an item that is part of a mixed dish is not reported, the mother did not observe all food items given to the child, or because of a lapse in memory. In previous studies among women, vegetables and fruits were generally more likely than other food groups to be misreported [38–40]. Another limitation of the data is that it does not provide information on other caregivers such as grandmothers who may also influence ZVF consumption. An in-depth analysis of household dynamics that may affect food consumption is not captured in this study, although the wealth quintile that was included in our analysis does take into account household size. Furthermore, our analysis did not account for the food environment, which determines food availability, access, utilization and feeding practices.

Despite these limitations, the new ZVF indicator is a useful tool for advocacy and monitoring diet quality among young children. Vegetable and fruit consumption is a critical component of young children’s diets and provides important micronutrients that support healthy growth and development.

Our study results indicate global action is needed to improve vegetable and fruit consumption among young children. Multidimensional strategies that address personal and external barriers to vegetable and fruit consumption for infants and young children are likely to be most effective [41, 42]. Interventions targeting the wider food system/agricultural production can work in tandem with school gardens, improved home gardens, cash transfers, nutrition education, and behavior change for families and communities [41, 43, 44]. As most research on effective strategies is from high income-settings, two areas for future research include generating evidence from LMICs on effective interventions and translating strategies from other contexts to improve vegetable and fruit consumption among young children.

## Supporting information

S1 Appendix (PDF)

## References

[1] WHO. Complementary feeding: report of the global consultation, and summary of guiding principles for complementary feeding of the breastfed child. Geneva, Switzerland; 2002.

[2] Thorne-Lyman AL, Shrestha M, Fawzi WW, Pasqualino M, Strand TA, Kvestad I, et al. Dietary Diversity and Child Development in the Far West of Nepal: A Cohort Study. Nutrients. 2019;11(8). doi: 10.3390/nu11081799 31382653 PMC6722734

[3] Arimond M, Ruel MT. Dietary Diversity Is Associated with Child Nutritional Status: Evidence from 11 Demographic and Health Surveys. The Journal of Nutrition. 2004;134(10):2579–85. doi: 10.1093/jn/134.10.2579 15465751

[4] Grimm KA, Kim SA, Yaroch AL, Scanlon KS. Fruit and vegetable intake during infancy and early childhood. Pediatrics. 2014;134 Suppl 1:S63–9. doi: 10.1542/peds.2014-0646K 25183758 PMC4258845

[5] Hodder RK, O’Brien KM, Stacey FG, Wyse RJ, Clinton-McHarg T, Tzelepis F, et al. Interventions for increasing fruit and vegetable consumption in children aged five years and under. Cochrane Database Syst Rev. 2018;5:CD008552. doi: 10.1002/14651858.CD008552.pub5 29770960 PMC6373580

[6] Hartley L, Igbinedion E, Holmes J, Flowers N, Thorogood M, Clarke A, et al. Increased consumption of fruit and vegetables for the primary prevention of cardiovascular diseases. Cochrane Database Syst Rev. 2013(6):CD009874. doi: 10.1002/14651858.CD009874.pub2 23736950 PMC6464871

[7] Gatica-Dominguez G, Neves PAR, Barros AJD, Victora CG. Complementary Feeding Practices in 80 Low- and Middle-Income Countries: Prevalence of and Socioeconomic Inequalities in Dietary Diversity, Meal Frequency, and Dietary Adequacy. J Nutr. 2021;151(7):1956–64. doi: 10.1093/jn/nxab088 33847352 PMC8245881

[8] Issaka AI, Agho KE, Page AN, Burns PL, Stevens GJ, Dibley MJ. Determinants of suboptimal complementary feeding practices among children aged 6–23 months in seven francophone West African countries. Matern Child Nutr. 2015;11 Suppl 1:31–52. doi: 10.1111/mcn.12193 26364790 PMC6860307

[9] Issaka AI, Agho KE, Page AN, Burns PL, Stevens GJ, Dibley MJ. Determinants of suboptimal complementary feeding practices among children aged 6–23 months in four anglophone West African countries. Matern Child Nutr. 2015;11 Suppl 1:14–30. doi: 10.1111/mcn.12194 26364789 PMC6860259

[10] Senarath U, Agho KE, Akram DE, Godakandage SS, Hazir T, Jayawickrama H, et al. Comparisons of complementary feeding indicators and associated factors in children aged 6–23 months across five South Asian countries. Matern Child Nutr. 2012;8 Suppl 1:89–106. doi: 10.1111/j.1740-8709.2011.00370.x 22168521 PMC6860856

[11] Oddo VM, Ickes SB. Maternal employment in low- and middle-income countries is associated with improved infant and young child feeding. Am J Clin Nutr. 2018;107(3):335–44. doi: 10.1093/ajcn/nqy001 29566201 PMC6248412

[12] Roess AA, Jacquier EF, Catellier DJ, Carvalho R, Lutes AC, Anater AS, et al. Food Consumption Patterns of Infants and Toddlers: Findings from the Feeding Infants and Toddlers Study (FITS) 2016. J Nutr. 2018;148(suppl_3):1525S–35S. doi: 10.1093/jn/nxy171 30247583 PMC6126630

[13] Wang H, Denney L, Zheng Y, Vinyes-Pares G, Reidy K, Wang P, et al. Food sources of energy and nutrients in the diets of infants and toddlers in urban areas of China, based on one 24-hour dietary recall. BMC Nutrition. 2015;1(1).

[14] Rodriguez-Ramirez S, Munoz-Espinosa A, Rivera JA, Gonzalez-Castell D, Gonzalez de Cosio T. Mexican Children under 2 Years of Age Consume Food Groups High in Energy and Low in Micronutrients. J Nutr. 2016;146(9):1916S–23S. doi: 10.3945/jn.115.220145 27511938

[15] Denney L, Afeiche MC, Eldridge AL, Villalpando-Carrion S. Food Sources of Energy and Nutrients in Infants, Toddlers, and Young Children from the Mexican National Health and Nutrition Survey 2012. Nutrients. 2017;9(5). doi: 10.3390/nu9050494 28505084 PMC5452224

[16] Denney L, Angeles-Agdeppa I, Capanzana MV, Toledo MB, Donohue J, Carriquiry A. Nutrient Intakes and Food Sources of Filipino Infants, Toddlers and Young Children are Inadequate: Findings from the National Nutrition Survey 2013. Nutrients. 2018;10(11). doi: 10.3390/nu10111730 30423865 PMC6267516

[17] Jacquier EF, Angeles-Agdeppa I, Lenighan YM, Toledo MB, Capanzana MV. Complementary feeding patterns of Filipino infants and toddlers lack diversity, especially among children from poor households. BMC Nutrition. 2020;6:51. doi: 10.1186/s40795-020-00376-1 33117553 PMC7586690

[18] Ricardo LIC, Gatica-Dominguez G, Neves PAR, Vaz JDS, Barros AJD, Wehrmeister FC. Sociodemographic inequalities in vegetables, fruits, and animal source foods consumption in children aged 6–23 months from 91 LMIC. Front Nutr. 2023;10:1046686. doi: 10.3389/fnut.2023.1046686 36866060 PMC9972219

[19] WHO & UNICEF. Indicators for assessing infant and young child feeding practices: definitions and measurement methods Geneva; 2021.

[20] Thorne-Lyman AL, Bevis LEM, Kuo H, Manohar S, Shrestha B, Kc A, et al. Season of Data Collection of Child Dietary Diversity Indicators May Affect Conclusions About Longer-Term Trends in Peru, Senegal, and Nepal. Curr Dev Nutr. 2021;5(8):nzab095. doi: 10.1093/cdn/nzab095 34466772 PMC8397594

[21] Croft TN, Marshall AMJ, Allen CK, al e. Guide to DHS Statistics. Rockville, Maryland, USA: ICF; 2018.

[22] Rutstein SO. The DHS wealth index: Approaches for rural and urban areas. Calverton, Maryland, USA: The Demographic Health Surveys Program. Macro International; 2008.

[23] Adler R, Wang J-J, Sapiano M, Huffman G, Chiu L, Xie PP, et al. Global Precipitation Climatology Project (GPCP) Climate Data Record (CDR), Version 2.3 (Monthly). In: Information NCfE, editor. 2016.

[24] Galway LP, Acharya Y, Jones AD. Deforestation and child diet diversity: A geospatial analysis of 15 Sub-Saharan African countries. Health Place. 2018;51:78–88. doi: 10.1016/j.healthplace.2018.03.002 29550735

[25] Gergel SE, Powell B, Baudron F, Wood SLR, Rhemtulla JM, Kennedy G, et al. Conceptual Links between Landscape Diversity and Diet Diversity: A Roadmap for Transdisciplinary Research. Bioscience. 2020;70(6):563–75. doi: 10.1093/biosci/biaa048 32665737 PMC7340543

[26] Niles MT, Emery BF, Wiltshire S, Brown ME, Fisher B, Ricketts TH. Climate impacts associated with reduced diet diversity in children across nineteen countries. Environmental Research Letters. 2021;16(1).

[27] Randell H, Grace K, Bakhtsiyarava M. Climatic Conditions and Infant Care: Implications for Child Nutrition in Rural Ethiopia. Popul Environ. 2021;42(4):524–52. doi: 10.1007/s11111-020-00373-3 34149138 PMC8210853

[28] Mayala B, Thomas D. Fish, David Eitelberg, Dontamsetti T. The DHS Program Geospatial Covariate Datasets Manual (Second Edition). Rockville, Maryland, USA: ICF; 2018.

[29] Baye K, Kennedy G. Estimates of dietary quality in infants and young children (6–23 mo): Evidence from demographic and health surveys of 49 low- and middle-income countries. Nutrition. 2020;78:110875. doi: 10.1016/j.nut.2020.110875 32653760

[30] Leroy JL, Ruel M, Verhofstadt E. The impact of conditional cash transfer programmes on child nutrition: a review of evidence using a programme theory framework. Journal of Development Effectiveness. 2009;1(2):103–29.

[31] Zhang Q, Alsuliman MA, Wright M, Wang Y, Cheng X. Fruit and Vegetable Purchases and Consumption among WIC Participants after the 2009 WIC Food Package Revision: A Systematic Review. Adv Nutr. 2020;11(6):1646–62. doi: 10.1093/advances/nmaa060 32452523 PMC7666910

[32] Erzse A, Goldstein S, Norris SA, Watson D, Kehoe SH, Barker M, et al. Double-duty solutions for optimising maternal and child nutrition in urban South Africa: a qualitative study. Public Health Nutr. 2021;24(12):3674–84. doi: 10.1017/S1368980020002426 32830637 PMC10195233

[33] WHO. The double burden of malnutrition. Geneva: World Health Organization; 2017.

[34] Wiggins S, Keats S. The rising cost of a health diet: Changing relative prices of foods in high-income emerging economies. Overseas Development Institute; 2015. Report No.: 2052–7209.

[35] Forestell CA, Mennella JA. Early determinants of fruit and vegetable acceptance. Pediatrics. 2007;120(6):1247–54. doi: 10.1542/peds.2007-0858 18055673 PMC2268898

[36] Chikhungu LC, Madise NJ. Seasonal variation of child under nutrition in Malawi: is seasonal food availability an important factor? Findings from a national level cross-sectional study. BMC Public Health. 2014;14:1146. doi: 10.1186/1471-2458-14-1146 25373873 PMC4246561

[37] Ruel MT. Measuring Infant and Young Child Complementary Feeding Practices: Indicators, Current Practice, and Research Gaps. Nestle Nutr Inst Workshop Ser. 2017;87:73–87. doi: 10.1159/000448939 28315900

[38] Hanley-Cook GT, Tung JYA, Sattamini IF, Marinda PA, Thong K, Zerfu D, et al. Minimum Dietary Diversity for Women of Reproductive Age (MDD-W) Data Collection: Validity of the List-Based and Open Recall Methods as Compared to Weighed Food Record. Nutrients. 2020;12(7). doi: 10.3390/nu12072039 32659995 PMC7400839

[39] Martin-Prevel Y, Becquey E, Arimond M. Food group diversity indicators derived from qualitative list-based questionnaire misreported some foods compared to same indicators derived from quantitative 24-hour recall in urban Burkina Faso. J Nutr. 2010;140(11):2086S–93S. doi: 10.3945/jn.110.123380 20881076

[40] Nguyen PH, Martin-Prevel Y, Moursi M, Tran LM, Menon P, Ruel MT, et al. Assessing Dietary Diversity in Pregnant Women: Relative Validity of the List-Based and Open Recall Methods. Curr Dev Nutr. 2020;4(1):nzz134. doi: 10.1093/cdn/nzz134 32258987 PMC7101484

[41] Kaur S. Barriers to consumption of fruits and vegetables and strategies to overcome them in low- and middle-income countries: a narrative review. Nutr Res Rev. 2022:1–28. doi: 10.1017/S0954422422000166 36004512

[42] Turner C, Kalamatianou S, Drewnowski A, Kulkarni B, Kinra S, Kadiyala S. Food Environment Research in Low- and Middle-Income Countries: A Systematic Scoping Review. Adv Nutr. 2020;11(2):387–97. doi: 10.1093/advances/nmz031 31079142 PMC7442349

[43] Harris J, Tan W, Raneri JE, Schreinemachers P, Herforth A. Vegetables for Healthy Diets in Low- and Middle-Income Countries: A Scoping Review of the Food Systems Literature. Food and Nutrition Bulletin. 2022;43(2):232–48. doi: 10.1177/03795721211068652 34991377 PMC9118491

[44] Harris J, van Zonneveld M, Achigan-Dako EG, Bajwa B, Brouwer ID, Choudhury D, et al. Fruit and vegetable biodiversity for nutritionally diverse diets: Challenges, opportunities, and knowledge gaps. Global Food Security. 2022;33.

