# Supplementary material for: Estimates and trends of zero vegetable or fruit consumption among children aged 6–23 months in 64 countries
Source: PLOS Glob Public Health. 2023 Jun 27;3(6):e0001662. doi: 10.1371/journal.pgph.0001662 (PMC10298791; doi:10.1371/journal.pgph.0001662)
Supplement: S1 Appendix — (PDF) [file pgph.0001662.s001.pdf]

Supporting Information

Table A. Description of sample and surveys included within West and Central Africa

Region and Country	Survey year	DHS phase	Total sample[1]	Don't know (%)	Missing (%)[2]	Included in analysis: Individual country prevalence	Regional prevalence	Trends	Multivariate
**West & Central Africa**									
Angola	2015-16	7	3,844	0.0	0.0	x	x		
Benin	2011-12	6	3,766	0.2	0.2			x	
Benin	2017-18	7	3,932	0.1	0.0	x	x	x	x
Burkina Faso	2010	6	4,233		0.1	x	x		x
Cameroon	2011	6	3,341	0.1	0.2			x	
Cameroon	2018-19	7	2,726	0.2	0.0	x	x	x	x
Chad	2014-15	6	4,589	0.0	0.4	x	x		x
Congo (Brazzaville)	2011-12	6	2,486	0.1	0.0	x	x		x
Congo Democratic Republic	2013-14	6	4,996	0.0	0.1	x	x		x
Cote d'Ivoire	2011-12	6	2,129	0.1	0.2	x	x		x
Gabon	2012	6	1,437	0.5	1.0	x	x		x
Gambia	2013	6	2,401	0.1	0.2			x	
Gambia	2019-20	7	2,132	0.2	0.0	x	x	x	x
Ghana	2008	5	835	0.0	0.3			x	
Ghana	2014	6	1,665	0.0	0.1	x	x	x	x
Guinea	2012	6	1,960	0.0	0.3			x	
Guinea	2018	7	1,925	0.2	0.0	x	x	x	x
Liberia	2006-07	5	1,482	0.3	0.5			x	
Liberia	2013	6	1,905	0.0	0.0			x	
Liberia	2019-20	7	1,394	0.0	0.0	x	x	x	x
Mali	2012-13	6	2,861	0.1	0.0			x	
Mali	2018	7	2,942	0.1	0.0	x	x	x	x
Niger	2012	6	3,500	0.1	0.2	x	x		x
Nigeria	2008	5	7,541	0.0	0.7			x	
Nigeria	2013	6	8,940	0.1	0.5			x	
Nigeria	2018	7	9,180	0.0	0.0	x	x	x	x
Sao Tome and Principe	2008-09	5	522	0.0	0.7	x			
Senegal	2010-11	6	3,203	0.1	0.0			x	
Senegal	2012-13	6	1,817	0.2	0.0				
Senegal	2014	6	1,722	0.3	0.0			x	
Senegal	2015	6	1,757	0.0	0.0				
Senegal	2016	6	1,700	0.1	0.0			x	x
Senegal	2017	7	3,264	0.1	0.0				
Senegal	2018	7	1,839	0.2	0.0				
Senegal	2019	7	1,678	0.3	0.0	x	x	x	
Sierra Leone	2008	5	1,581	0.3	1.3			x	
Sierra Leone	2013	6	3,169	0.1	0.9			x	
Sierra Leone	2019	7	2,640	0.0	0.0	x	x	x	x
Togo	2013-14	6	2,022	0.0	0.2	x	x		

1 Weighted sample
2 Missing responses were those that missing for all possible questions on fruit and vegetable consumption (estimate unweighted)
3 Don't know responses were those where the respondent answered "Don't know" to all possible questions on fruit and vegetable consumption (estimate unweighted)

Table B. Description of sample and surveys included within South and East Africa

Region and Country	Survey year	DHS phase	Total sample[1]	Don't know (%)[3]	Missing (%)[2]	Included in analysis: Individual country prevalence	Regional prevalence	Trends	Multivariate
**South & East Africa**									
Burundi	2010-11	6	2,281	0.0	0.0			x	
Burundi	2016-17	7	4,073	0.0	0.0	x	x	x	x
Comoros	2012	6	941	0.2	0.3	x	x		x
Eswatini	2006-07	5	777	0.0	0.8	x			
Ethiopia	2011	6	2,995	0.1	0.1			x	
Ethiopia	2016	7	3,029	0.1	0.0			x	x
Ethiopia	2019	7	1,504	0.1	0.0	x	x	x	
Kenya	2008-09	5	1,654	0.1	0.1			x	
Kenya	2014	6	2,639	0.0	0.0	x	x	x	x
Lesotho	2009-10	5	1,036	0.4	0.0			x	
Lesotho	2014	6	937	1.1	0.0	x	x	x	x
Madagascar	2008-09	5	3,496	0.0	0.4	x			
Malawi	2010	5	5,674	0.0	0.4			x	
Malawi	2015-16	7	4,749	0.0	0.0	x	x	x	x
Mozambique	2011	6	3,494	0.3	0.0	x	x		x
Namibia	2006-07	5	1,365	0.2	0.1			x	
Namibia	2013	6	1,254	0.0	0.4	x	x	x	x
Rwanda	2010-11	6	2,412	0.0	0.1			x	
Rwanda	2014-15	6	2,444	0.0	0.1	x	x	x	x
South Africa	2016	7	875	0.7	0.0	x	x		x
Tanzania	2009-10	5	2,324	0.0	0.3			x	
Tanzania	2015-16	7	2,995	0.1	0.0	x	x	x	x
Uganda	2006	5	2,296	0.1	0.1			x	
Uganda	2011	6	2,212	0.2	0.1			x	
Uganda	2016	7	4,223	0.0	0.0	x	x	x	x
Zambia	2007	5	1,861	0.1	0.1			x	
Zambia	2013-14	6	3,755	0.1	0.3			x	
Zambia	2018-19	7	2,753	0.1	0.0	x	x	x	x
Zimbabwe	2005-06	5	1,488	0.1	0.4			x	
Zimbabwe	2010-11	6	1,621	0.4	0.0			x	
Zimbabwe	2015	7	1,696	0.5	0.0	x	x	x	x

1 Weighted sample
2 Missing responses were those that missing for all possible questions on fruit and vegetable consumption (estimate unweighted)
3 Don't know responses were those where the respondent answered "Don't know" to all possible questions on fruit and vegetable consumption (estimate unweighted)

Table C. Description of sample and surveys included within Eastern Mediterranean and Europe

Region and Country	Survey year	DHS phase	Total sample[1]	Don't know (%)[3]	Missing (%)[2]	Included in analysis: Individual country prevalence	Regional prevalence	Trends	Multivariate
**Eastern Mediterranean & Europe**									
Afghanistan	2015	7	8,262	0.0	0.4	x	x		
Albania	2008-09	5	376	0.0	0.0			x	
Albania	2017-18	7	768	0.0	0.0	x	x	x	x
Armenia	2010	6	470	0.0	0.6			x	
Armenia	2015-16	7	521	0.2	0.0	x	x	x	x
Azerbaijan	2006	5	666	0.0	0.6	x			
Egypt	2008	5	3,365	0.1	0.1			x	
Egypt	2014	6	4,890	0.1	0.0	x	x	x	x
Jordan	2007	5	2,730	0.1	0.0			x	
Jordan	2012	6	2,741	0.0	0.0			x	
Jordan	2017-18	7	2,517	0.0	0.0	x	x	x	x
Kyrgyz Republic	2012	6	1,285	0.0	0.2	x	x		x
Tajikistan	2012	6	1,624	0.0	0.4			x	
Tajikistan	2017	7	1,924	0.1	0.0	x	x	x	x
Yemen	2013	6	4,463	0.2	0.2	x	x		

1 Weighted sample
2 Missing responses were those that missing for all possible questions on fruit and vegetable consumption (estimate unweighted)
3 Don't know responses were those where the respondent answered "Don't know" to all possible questions on fruit and vegetable consumption (estimate unweighted)

Table D. Description of sample and surveys included within South-East Asia and Western Pacific

Region and Country	Survey year	DHS phase	Total sample[1]	Don't know (%)[3]	Missing (%)[2]	Included in analysis: Individual country prevalence	Regional prevalence	Trends	Multivariate
**South-East Asia**									
Bangladesh	2007	5	1,744	0.0	0.0			x	
Bangladesh	2011	6	2,374	0.1	0.2			x	
Bangladesh	2014	6	2,458	0.0	0.3			x	x
Bangladesh	2017-18	7	2,447	0.1	0.0	x	x	x	
Cambodia	2005-06	5	2,257	0.2	0.0			x	
Cambodia	2010-11	5	2,399	0.1	0.2			x	
Cambodia	2014	6	2,175	0.1	0.0	x	x	x	x
India	2005-06	5	15,468	0.1	0.2			x	
India	2015-16	6	71,326	0.0	0.0	x	x	x	x
Indonesia	2007	5	4,692	0.1	0.2			x	
Indonesia	2012	6	5,059	0.1	1.2			x	
Indonesia	2017	7	4,940	0.1	0.6	x	x	x	
Maldives	2009	5	1,249	0.0	0.9			x	
Maldives	2016-17	7	783	0.2	0.0	x	x	x	
Myanmar	2015-16	7	1,237	0.1	0.0	x	x		x
Nepal	2006	5	1,464	0.0	0.0			x	
Nepal	2011	6	1,476	0.0	0.0			x	
Nepal	2016	7	1,489	0.0	0.0	x	x	x	x
Pakistan	2012-13	6	3,015	0.0	0.2			x	
Pakistan	2017-18	7	2,763	0.0	0.0	x	x	x	x
Papua New Guinea	2016-18	7	2,648	0.2	1.9	x	x		
Philippines	2008	5	1,844	0.3	0.1	x			
Timor-Leste	2009-10	5	2,632	0.0	0.0			x	
Timor-Leste	2016	7	2,081	0.0	0.0	x	x	x	x

1 Weighted sample
2 Missing responses were those that missing for all possible questions on fruit and vegetable consumption (estimate unweighted)
3 Don't know responses were those where the respondent answered "Don't know" to all possible questions on fruit and vegetable consumption (estimate unweighted)

Table E. Description of sample and surveys included within Latin America and the Caribbean

Region and Country	Survey year	DHS phase	Total sample[1]	Don't know (%)[3]	Missing (%)[2]	Included in analysis: Individual country prevalence	Regional prevalence	Trends	Multivariate
**Latin America & Caribbean**									
Bolivia	2008	5	2,543	0.0	0.2	x			
Colombia	2009-10	5	4,571	1.0	1.7	x	x		x
Dominican Republic	2007	5	2,875	0.2	0.8			x	
Dominican Republic	2013	6	1,052	0.6	0.2	x	x	x	x
Guatemala	2014-15	6	3,589	0.0	0.0	x	x		x
Guyana	2009	5	520	0.0	1.6	x	x		x
Haiti	2005-06	5	1,627	0.0	0.0			x	
Haiti	2012	6	1,916	0.0	0.3			x	
Haiti	2016-17	7	1,577	0.0	0.0	x	x	x	x
Honduras	2005-06	5	2,909	0.4	0.1			x	
Honduras	2011-12	6	3,071	0.2	0.1	x	x	x	x
Peru	2007-08	5	2,404	0.1	0.1			x	
Peru	2009	6	2,789	0.4	0.0				
Peru	2010	6	2,524	0.1	0.0			x	
Peru	2011	6	2,377	0.3	0.0				
Peru	2012	6	2,599	0.3	0.0	x	x	x	

1 Weighted sample

2 Missing responses were those that missing for all possible questions on fruit and vegetable consumption (estimate unweighted)

3 Don't know responses were those where the respondent answered "Don't know" to all possible questions on fruit and vegetable consumption (estimate unweighted)

Table F. Percent of children aged 6-23 months with zero vegetable or fruit consumption in the most recent Demographic and Health Surveys in West and Central Africa

Region and Country	Year	Prevalence (%)	95% CI	N
**West and Central Africa**	**2010-2020**	**56.1**	**[55.4-56.9]**	**60,317**
Angola	2015-16	38.9	[36.4-41.4]	3,844
Benin	2017-18	59.3	[57.2-61.5]	3,932
Burkina Faso	2010	75.0	[73.1-76.7]	4,233
Cameroon	2018-19	38.7	[36.0-41.4]	2,726
Chad	2014-15	71.2	[68.9-73.4]	4,589
Congo (Brazzaville)	2011-12	50.1	[46.4-53.7]	2,486
Congo Democratic Republic	2013-14	30.4	[28.1-32.8]	4,996
Cote d'Ivoire	2011-12	76.2	[73.6-78.6]	2,129
Gabon	2012	55.5	[51.7-59.2]	1,437
Gambia	2019-20	64.9	[61.6-68.0]	2,132
Ghana	2014	51.4	[48.0-54.7]	1,666
Guinea	2018	64.2	[61.4-67.0]	1,926
Liberia	2019-20	57.4	[53.0-61.7]	1,394
Mali	2018	57.2	[54.4-59.9]	2,942
Niger	2012	67.9	[65.2-70.5]	3,500
Nigeria	2018	54.5	[53.0-56.0]	9,180
Sao Tome and Principe	2008-09	27.9	[22.2-34.3]	522
Senegal	2019	54.9	[51.1-58.6]	1,678
Sierra Leone	2019	53.5	[51.1-55.8]	2,640
Togo	2013-14	45.4	[42.5-48.4]	2,022

Table G. Percent of children aged 6-23 months with zero vegetable or fruit consumption in the most recent Demographic and Health Surveys in South and East Africa

Region and Country	Year	Prevalence (%)	95% CI	N
**South and East Africa**	**2011-2019**	39.5	**[38.4-40.5]**	**34,571**
Burundi	2016-17	18.2	[16.8-19.8]	4,073
Comoros	2012	55.6	[50.5-60.7]	941
Eswatini	2006-07	28.3	[25.0-31.8]	777
Ethiopia	2019	70.5	[65.8-74.9]	1,504
Kenya	2014	30.2	[28.0-32.5]	2,639
Lesotho	2014	51.1	[47.4-54.8]	937
Madagascar	2008-09	35.1	[32.7-37.6]	3,496
Malawi	2015-16	25.3	[23.7-27.0]	4,749
Mozambique	2011	36.7	[34.6-38.9]	3,494
Namibia	2013	52.8	[49.3-56.2]	1,254
Rwanda	2014-15	26.3	[24.5-28.3]	2,444
South Africa	2016	39.9	[35.7-44.3]	875
Tanzania	2015-16	31.9	[29.5-34.4]	2,995
Uganda	2016	46.3	[44.3-48.4]	4,223
Zambia	2018-19	32.5	[30.1-34.9]	2,753
Zimbabwe	2015	34.8	[32.1-37.7]	1,696

Table H. Percent of children aged 6-23 months with zero vegetable or fruit consumption in the most recent Demographic and Health Surveys in Eastern Mediterranean and Europe

Region and Country	Year	Prevalence (%)	95% CI	N
**Eastern Mediterranean and Europe**	**2012-2018**	**48.3**	**[46.9-49.7]**	**25,154**
Afghanistan	2015	60.8	[57.3-64.2]	8,262
Albania	2017-18	29.1	[24.7-33.9]	768
Armenia	2015-16	26.5	[22.5-30.9]	521
Azerbaijan	2006	43.0	[38.1-48.1]	666
Egypt	2014	46.3	[44.6-48.1]	4,890
Jordan	2017-18	45.5	[42.6-48.4]	2,517
Kyrgyz Republic	2012	49.4	[45.9-52.9]	1,285
Tajikistan	2017	61.3	[58.2-64.3]	1,924
Yemen	2013	67.6	[65.4-69.6]	4,463

Table I. Percent of children aged 6-23 months with zero vegetable or fruit consumption in the most recent Demographic and Health Surveys in South-East Asia and Western Pacific

Region and Country	Year	Prevalence (%)	95% CI	N
**South-East Asia and Western Pacific**	**2014-2018**	**39.7**	**[38.6-40.7]**	**94,842**
Bangladesh	2017-18	49.3	[46.8-51.8]	2,447
Cambodia	2014	36.3	[33.7-39.0]	2,175
India	2015-16	55.5	[55.0-56.1]	71,326
Indonesia	2017	19.5	[18.1-20.8]	4,940
Maldives	2016-17	18.7	[14.8-23.2]	783
Myanmar	2015-16	56.4	[52.9-59.8]	1,237
Nepal	2016	40.1	[37.0-43.4]	1,489
Pakistan	2017-18	64.2	[61.2-67.1]	2,763
Papua New Guinea	2016-18	17.7	[15.5-20.2]	2,648
Philippines	2008	24.6	[22.3-27.0]	1,844
Timor-Leste	2016	39.0	[36.0-42.1]	2,081

Table J. Percent of children aged 6-23 months with zero vegetable or fruit consumption in the most recent Demographic and Health Surveys in Latin America and the Caribbean and Global region

Region and Country	Year	Prevalence (%)	95% CI	N
**Latin America and the Caribbean**	**2009-2017**	**34.5**	**[33.3-35.7]**	**18,124**
Bolivia	2008	21.7	[19.7-23.9]	2,543
Colombia	2009-10	30.6	[29.0-32.3]	4,571
Dominican Republic	2013	40.0	[36.2-43.9]	1,053
Guatemala	2014-15	28.4	[26.6-30.3]	3,589
Guyana	2009	33.2	[28.7-38.0]	520
Haiti	2016-17	56.1	[52.9-59.2]	1,577
Honduras	2011-12	37.3	[35.1-39.6]	3,071
Peru	2012	15.6	[13.9-17.4]	2,599
**Global**	**2009-2020**	**45.7**	**[45.2-46.2]**	**235,091**

Table K. Percent of children aged 6-23 months with zero vegetable or fruit consumption in trends analysis, by world region and country

	Survey number:			
**Region and Country**	**1**	**2[1]**	**3[1]**	**4[1]**
**West and Central Africa**				
Benin	53.7***▲	59.3***▲		
Cameroon	41.4	38.7▼		
Gambia	76.3	64.9***▼		
Ghana	30.7	51.4***▲		
Guinea	82.5	64.2***▼		
Liberia	53.8	58▲	57▼	
Mali	68.2***▼	57***▼		
Nigeria	47.9	61***▲	54***▼	
Senegal	51.0	53.6▲	67***▲	55***▼
Sierra Leone	38.5	61.5***▲	53***▼	
**South and East Africa**				
Burundi	20.2	18.2▼		
Ethiopia	82.9	70.1***▼	70.5▲	
Kenya	26.7	66.3***▲		
Lesotho	33.6	51.1***▲		
Malawi	32.7	25.3***▼		
Namibia	50.5	52.8▲		
Rwanda	28.3	26.3▼		
Tanzania	29.9	31.9▲		
Uganda	53.1	52▼	46.3** ▼	
Zambia	26.3	36.5***▲	32.5*▼	
Zimbabwe	54.8	39.7***▼	34.8*▼	
**Eastern Mediterranean and Europe**				
Albania	26.0	29.1▲		
Armenia	26.9	26.5▼		
Egypt	47.9	46.3▼		
Jordan	25.6	33***▲	45.5***▲	
Tajikistan	51.9	61.3***▲		
**South-East Asia and Western Pacific**				
Bangladesh	30.1	56.1***▲	53.3▼	49.3*▼
Cambodia	39.5	37.4▼	36.3▼	
India	60.1	55.5***▼		
Indonesia	19.5	23.2** ▲	19.5** ▼	
Maldives	32.3	18.7***▼		
Nepal	43.2	56.4***▲	40.1***▼	
Pakistan	58.5	64.2** ▲		
Timor-Leste	36.5	39▲		
**Latin America and the Caribbean**				
Dominican Republic	33.5	40** ▲		
Haiti	48.6	40.4***▼	56.1***▲	
Honduras	45.5	37.3***▼		
Peru	17.1	15.9▼	15.6▼	

1 The surveys included in the trend are numbered in chronological order sequentially, starting with the oldest survey as 1. Asterisks indicate if survey had a significant change from previous survey *p<0.05, **p<0.01, ***p<0.001 and the shape indicates an increase (▲) or decrease (▼) in ZVF compared to the previous survey

Table L. Study sample description used in multivariate analysis, by world region

	Global	West & Central Africa	South & East Africa	Eastern Mediterranean & Europe	South-East Asia & Western Pacific	Latin America & the Caribbean
	%	%	%	%	%	%
**Child's characteristics**						
Birth order						
First born	28.2%	21.8%	26.4%	33.2%	35.3%	35.4%
Second - fourth	51.1%	48.2%	50.0%	59.8%	52.5%	50.8%
Fifth +	20.7%	30.0%	23.6%	7.0%	12.2%	13.8%
Sex of child						
Male	50.9%	50.9%	50.0%	51.7%	52.2%	50.8%
Female	49.1%	49.1%	50.0%	48.3%	47.8%	49.2%
Age in months, mean(SD)	14.2(5.1)	14.1(5.1)	14.2(5.1)	14.2(5.2)	14.4(5.1)	14.3(5.1)
Breastfeeding						
Not currently	27.7%	22.0%	24.0%	44.1%	21.4%	40.4%
Currently	72.3%	78.0%	76.0%	55.9%	78.6%	59.6%
**Mother's characteristics**						
Education						
None, primary	55.7%	70.5%	64.1%	15.2%	51.1%	45.4%
Secondary, higher	44.3%	29.5%	35.9%	84.8%	48.9%	54.6%
Employment		30.5%				
Not working	47.5%	69.5%	41.7%	81.4%	63.2%	49.7%
Working	52.5%		58.3%	18.6%	36.8%	50.3%
Media exposure						
None	42.0%	48.5%	48.7%	10.7%	45.5%	31.7%
Some	58.0%		51.2%	89.3%	54.5%	68.3%
Mother's age						
<24	9.2%	9.9%	9.3%	2.6%	8.2%	14.0%
25-24	74.1%	70.7%	72.3%	83.4%	80.0%	71.5%
35+	16.7%	19.4%	18.4%	14.0%	11.8%	14.5%
**Household & cluster characteristics**						
Wealth						
Lowest	23.1%	22.3%	23.9%	20.1%	22.7%	26.8%
Second	21.6%	21.9%	21.5%	21.1%	20.7%	22.3%
Middle	20.4%	20.3%	20.1%	22.2%	20.2%	20.2%
Fourth	18.9%	19.2%	18.5%	20.1%	19.2%	17.0%
Highest	16.1%	16.2%	16.1%	16.6%	17.3%	13.7%
Residence						
Urban	37.5%	40.6%	27.7%	46.9%	29.6%	46.5%
Rural	62.5%	59.4%	72.3%	53.1%	70.4%	53.5%
Rainy season						
No	63.3%	60.2%	52.1%	89.0%	82.2%	72.3%
Yes	36.7%	39.8%	47.9%	11.0%	17.8%	27.7%

Table M. Unadjusted odds ratios of bivariate analysis, by world region

	Global		West and Central Africa		South and East Africa		Eastern Mediterranean and Europe		South-East Asia and Western Pacific		Latin America and the Caribbean	
	AOR	95% CI	AOR	95% CI	AOR	95% CI	AOR	95% CI	AOR	95% CI	AOR	95% CI
**Child's characteristics**												
Birth order (ref: First born)												
Second - fourth	1.03	0.99 - 1.07	0.97	0.91 - 1.04	0.99	0.92 - 1.07	0.93	0.82 - 1.06	1.03	0.95 - 1.12	1.03	0.92 - 1.15
Fifth +	1.24***	1.19 - 1.30	0.99	0.92 - 1.06	1.04	0.95 - 1.13	1.29*	1.05 - 1.58	1.26***	1.11 - 1.43	1.56***	1.34 - 1.81
Sex of child (ref: Male)												
Female	0.98	0.95 - 1.02	1.00	0.95 - 1.06	0.95	0.90 - 1.01	0.96	0.85 - 1.08	1.00	0.92 - 1.09	1.00	0.90 - 1.12
Age	0.91***	0.91 - 0.92	0.91***	0.90 - 0.91	0.92***	0.91 - 0.93	0.92***	0.91 - 0.93	0.89***	0.89 - 0.90	0.94***	0.93 - 0.95
Breastfeeding (ref: Not currently)												
Currently	1.56***	1.50 - 1.62	1.66***	1.55 - 1.77	1.17***	1.09 - 1.27	1.72***	1.52 - 1.93	1.44***	1.30 - 1.59	1.24***	1.10 - 1.39
Breastfeeding x Age												
Interaction	0.91***	0.91 - 0.92	0.90***	0.90 - 0.91	0.91***	0.90 - 0.91	0.93***	0.91 - 0.94	0.89***	0.88 - 0.90	0.94***	0.93 - 0.95
**Mother's characteristics**												
Education (ref: None, primary)												
Secondary, higher	0.69***	0.66 - 0.71	0.70***	0.66 - 0.75	0.89**	0.83 - 0.96	0.95	0.80 - 1.14	0.70***	0.64 - 0.76	0.62***	0.55 - 0.69
Employment (ref: Not working)												
Working	0.81***	0.78 - 0.84	0.77***	0.73 - 0.82	0.55***	0.52 - 0.59	0.68***	0.57 - 0.81	0.66***	0.61 - 0.73	0.80***	0.72 - 0.89
Media exposure (ref: None)												
Some	0.75***	0.72 - 0.77	0.91***	0.86 - 0.96	0.78***	0.73 - 0.83	0.63***	0.50 - 0.80	0.76***	0.69 - 0.83	0.79***	0.71 - 0.88
Mother's age (ref: <24)												
25-34	0.87***	0.82 - 0.92	0.88**	0.81 - 0.95	0.84**	0.75 - 0.93	0.72*	0.53 - 0.99	0.82**	0.71 - 0.95	0.85*	0.72 - 0.99
35+	0.84***	0.79 - 0.89	0.79***	0.72 - 0.86	0.77***	0.69 - 0.87	0.60**	0.43 - 0.85	0.85	0.72 - 1.02	0.92	0.74 - 1.13
**Household and cluster characteristics**												
Wealth (ref: Lowest)												
Second	0.92***	0.88 - 0.97	0.98	0.91 - 1.05	0.88**	0.80 - 0.96	0.92	0.78 - 1.08	0.95	0.84 - 1.06	0.80**	0.69 - 0.92
Middle	0.85***	0.81 - 0.90	0.91*	0.84 - 0.99	0.82***	0.75 - 0.91	0.82*	0.70 - 0.97	0.87*	0.77 - 0.99	0.69***	0.60 - 0.81
Fourth	0.79***	0.75 - 0.83	0.93	0.86 - 1.02	0.70***	0.63 - 0.78	0.78**	0.66 - 0.94	0.78***	0.69 - 0.89	0.52***	0.44 - 0.62
Highest	0.64***	0.61 - 0.68	0.80***	0.72 - 0.87	0.57***	0.51 - 0.64	0.58***	0.47 - 0.71	0.66***	0.57 - 0.75	0.37***	0.30 - 0.45
Residence (ref: Urban)												
Rural	1.27***	1.22 - 1.32	1.20***	1.13 - 1.28	1.31***	1.20 - 1.42	1.42***	1.25 - 1.60	1.22***	1.11 - 1.35	1.36***	1.22 - 1.51
Rainy season (ref: No)												
Yes	0.88***	0.85 - 0.92	0.61***	0.57 - 0.64	0.85***	0.79 - 0.92	0.92	0.72 - 1.16	1.02	0.91 - 1.15	1.07	0.94 - 1.21
Mean temperature	1.00	1.00 - 1.00	1.00	1.00 - 1.00	1.00*	1.00 - 1.00	1.00***	1.00 - 1.00	1.00*	1.00 - 1.00	1.00	1.00 - 1.00
Vegetation index [1]	1.02*	1.00 - 1.04	1.00	0.97 - 1.04	1.03	0.99 - 1.07	1.12***	1.05 - 1.20	1.03	0.98 - 1.08	1.08**	1.02 - 1.14
Altitude [2]	1.00*	1.00 - 1.00	1.00	1.00 - 1.00	1.00***	1.00 - 1.00	1.00	1.00 - 1.00	1.00	1.00 - 1.00	1.00*	1.00 - 1.00

Note: Asterisks indicate the p-value *p<0.05, **p<0.01, ***p<0.001. AOR - Adjusted Odds Ratio. CI - Confidence Interval. 'Ref' – reference category. 1 Unitless index. Higher scores indicate higher vegetation vigor/photosynthetic activity, 2 Degrees of elevation
